# Identification of Myelin Basic Protein Proximity Interactome Using TurboID Labeling Proteomics

**DOI:** 10.3390/cells12060944

**Published:** 2023-03-20

**Authors:** Evgeniya V. Smirnova, Tatiana V. Rakitina, Rustam H. Ziganshin, George A. Saratov, Georgij P. Arapidi, Alexey A. Belogurov, Anna A. Kudriaeva

**Affiliations:** 1 Shemyakin and Ovchinnikov Institute of Bioorganic Chemistry, Russian Academy of Sciences, 117997 Moscow, Russia; 2 Phystech School of Biological and Medical Physics, Moscow Institute of Physics and Technology (National Research University), 141701 Dolgoprudny, Russia; 3 Federal Research and Clinical Center of Physical-Chemical Medicine of Federal Medical Biological Agency, 119435 Moscow, Russia; 4 Department of Biological Chemistry, Evdokimov Moscow State University of Medicine and Dentistry, Ministry of Health of Russian Federation, 127473 Moscow, Russia

**Keywords:** myelin basic protein, multiple sclerosis, proximity labeling proteomics, immunoprecipitation, membrane

## Abstract

Myelin basic protein (MBP) is one of the key structural elements of the myelin sheath and has autoantigenic properties in multiple sclerosis (MS). Its intracellular interaction network is still partially deconvoluted due to the unfolded structure, abnormally basic charge, and specific cellular localization. Here we used the fusion protein of MBP with TurboID, an engineered biotin ligase that uses ATP to convert biotin to reactive biotin-AMP that covalently attaches to nearby proteins, to determine MBP interactome. Despite evident benefits, the proximity labeling proteomics technique generates high background noise, especially in the case of proteins tending to semi-specific interactions. In order to recognize unique MBP partners, we additionally mapped protein interaction networks for deaminated MBP variant and cyclin-dependent kinase inhibitor 1 (p21), mimicking MBP in terms of natively unfolded state, size and basic amino acid clusters. We found that in the plasma membrane region, MBP is colocalized with adhesion proteins occludin and myelin protein zero-like protein 1, solute carrier family transporters ZIP6 and SNAT1, Eph receptors ligand Ephrin-B1, and structural components of the vesicle transport machinery—synaptosomal-associated protein 23 (*SNAP23*), vesicle-associated membrane protein 3 (*VAMP3*), protein transport protein hSec23B and cytoplasmic dynein 1 heavy chain 1. We also detected that MBP potentially interacts with proteins involved in Fe^2+^ and lipid metabolism, namely, ganglioside GM2 activator protein, long-chain-fatty-acid-CoA ligase 4 (*ACSL4*), NADH-cytochrome b5 reductase 1 (*CYB5R1*) and metalloreductase STEAP3. Assuming the emerging role of ferroptosis and vesicle cargo docking in the development of autoimmune neurodegeneration, MBP may recruit and regulate the activity of these processes, thus, having a more inclusive role in the integrity of the myelin sheath.

## 1. Introduction

Myelin basic protein (MBP) is an important structural unit of the myelin sheath of axons and is associated with many neurodegenerative diseases, in particular, multiple sclerosis (MS) [1,2,3]. MS is an autoimmune, multifactorial, demyelinating and neurodegenerative disease of unknown pathogenesis [4]. Inflammation in the CNS during MS causes demyelination of axons leading to its damage and subsequent neuron death. The etiology of MS is believed to be triggered by myelin-reactive self-activation of the immune response in genetically susceptible people [5].

MBP, which was identified in the early 1960s [6], is the second most abundant myelin protein. It represents about 30% of the total CNS myelin proteins and is the most widely studied myelin protein in relation to MS. MBP is an intrinsically disordered protein (IDP) [7,8] that does not have a well-defined globular structure. Its conformation may significantly change depending on the environment and surrounding interactors. Indeed, MBP forms ⍺-helical structures and tightly attach to the membrane upon binding to negatively charged lipids on the cytoplasmic surface of the myelin sheath and, thus, causing adhesion [7,9,10]. The disordered nature of MBP suggests that it may be a multifunctional protein [11]. MBP binds to calmodulin [12,13,14], actin [13,14,15], tubulin [16,17], and SH3-domain-containing proteins [18]. It causes actin polymerization and binding [13,14,15] and also tethers actin filaments and the SH3 domain of Fyn tyrosine kinase to lipid bilayers [13,18,19,20,21]. It has also been shown that MBP can couple microtubules with the lipid bilayer and actin filaments [22]. Thus, MBP can serve as a scaffold protein, which recruits other proteins to the cytoskeleton and to the cytoplasmic surface of the plasma membrane.

The study of the protein environment of MBP is complicated by its high degree of internal disorder and small size. In addition, MBP is present in an organism as a large set of isoforms and modifications [23]. One of these modifications, the deimination of arginine amino acid residues, leads to the formation of uncharged citrulline-containing forms of MBP and is associated with MS [24,25]. Therefore, when studying the protein environment of MBP, it is necessary to consider the high degree of nonspecific interactions due to both structural variability and post-translational modifications, as well as the effects associated with the exogenous MBP overexpression. Previously, using the cross-linking method, we identified a number of proteins closely related to MBP, including those involved in MBP biogenesis, cytoskeleton regulation, cell adhesion, protein traffic and degradation. While the interaction of MBP with the b-hairpin C-terminal peptide of integral transmembrane protein II associated with familial British and Danish dementia (Bri2) was detected using a yeast two-hybrid system [26,27].

Here we comprehensively studied MBP Interactome utilizing proximity labeling proteomics technique (TurboID) and ordinary FLAG-tag-based immunoprecipitation. Proximity labeling is carried out using enzymes that catalyze the conversion of an inert low molecular weight substrate into a highly reactive and short-lived diffusible intermediate. This reactive molecule, usually conjugated to an affinity tag, such as biotin, diffuses from the active site of the enzyme and non-specifically covalently labels nearby endogenous biomolecules.

Since covalent labeling is performed in living cells with maintaining molecular complexes, cell membranes, and compartments, thus, spatial relationships and interaction networks are preserved in their original state. The covalent modification provides a unique chemical label, which can then be used for selective enrichment at the protein level (e.g., using streptavidin-conjugated beads) or at the peptide level (e.g., using anti-biotin antibody beads [28]), as well as for subsequent identification of labeled molecules. Proximity labeling has been shown to be effective for various types of biomolecules, including RNA and DNA [29,30], but this method has proved to be the most reliable for cellular proteins. In the case of proteins, quantitative mass spectrometry has provided the technological possibility of accurate, sensitive, and reproducible proteomic analysis [31,32,33].

Over the past few years, a diverse array of enzymes and labels has been developed. Prominent among these are biotin ligase-based approaches that do not require toxic reagents but instead simply utilize the highly soluble and nontoxic substrate biotin, while ATP is provided by cells. Biotin ligases adenylate biotin to form a reactive intermediate, biotin adenosine monophosphate (biotin-5′-AMP), which diffuses from the enzyme active site and reacts with the lysine side chain amines of nearby proteins [34,35]. It has been experimentally established that the radius of labeling with biotin-5′-AMP generated by biotin ligase BirA in living cells is ~10 nm [36], which makes it possible to biotinylate only adjacent proteins [37]. Unlike the first versions of engineered enzymes based on bacterial biotin ligases, such as BioID biotin, ligase originates from *Escherichia coli,* biotin ligase (BirA) having an extremely low activity (more than 18 h of labeling is required) [35,36,38], more recent specifically modified version, TurboID, has faster labeling kinetics (less than 10 min) and is ideal for *in vivo* applications [39].

To exclude nonspecific interaction, we used both wild-type MBP and “deaminated” MBP as target proteins and additionally implemented a cyclin-dependent kinase inhibitor 1 (p21), which is similar to MBP in structural properties but not related to its functionality. As a result, we observed novel modalities of MBP in vesicle cargo docking and suggested its possible role in lipid metabolism and ferroptosis.

## 2. Materials and Methods

### 2.1. Cells and Transfection

HEK293T cells were obtained from the Russian Cell Culture Collection (RCCC, Institute of Cytology of the Russian Academy of Sciences, St-Petersburg, Russia). HEK293T cells were maintained by a passage in Dulbecco’s modified Eagle’s medium supplemented with 100 μg/mL streptomycin, 100 units/mL penicillin, and 10% fetal bovine serum (FBS) (pH 7.2–7.4) in a humidified atmosphere containing 5% CO_2_ at 37 °C. Fresh HEK293T cells were plated on 6-well plates pre-coated with human fibronectin and grown to confluence. Then the cells were transfected with the MBP_Flag_TurboID, MBPCit_Flag_TurboID, p21_Flag_TurboID, and Flag_TurboID (as negative control) expression plasmids using Lipofectamine LTX Reagent with PLUS Reagent (Thermo Fisher Scientific, Waltham, MA, USA) according to the manufacturer’s instructions. All the labeling, immunocytochemistry, and immunoprecipitation experiments were conducted at 24 or 48 h after transfection.

### 2.2. Proximity Labeling in Mammalian Cells with TurboID and Preparation of Proteomic Samples

At 24 h after transfection with MBP_Flag_TurboID, MBPCit_Flag_TurboID, p21_Flag_TurboID, and Flag_TurboID constructs, the medium over transfected HEK293T cells was changed to a new with 50 μM biotin. The reaction was stopped after 30 min of labeling by washing with ice-cold PBS. Next, the cell pellets were lysed using RIPA buffer containing 50 mM Tris-HCl pH7.5, 150 mM NaCl, 0.1% SDS, 0.5% sodium deoxycholate, and 1% Triton X-100. The lysates were clarified by centrifugation at 13,000× *g* for 20 min at 4 °C. The supernatant was incubated with Streptavidin-agarose (Thermo Fisher Scientific, Waltham, MA, USA) resin (pre-washed with RIPA buffer) overnight at 4 °C with constant rotation. Next, the resin was centrifuged at 2000 g for 2 min at 4 °C. The resin was washed twice with 1 mL of RIPA buffer, once with 1 mL of 1 M KCl (incubation for 2 min at RT), then once with 0.1 M Na_2_CO_3_ (incubation for 10 s at RT), then twice in a buffer containing 10 mM Tris-HCl pH 8.0 and 2 M urea (incubation for 10 s at RT) and, finally, washed twice with 1 mL of RIPA buffer. The supernatant was removed, and the resin was resuspended in 100 µL of 1× Sample buffer with 2 mM biotin and 20 mM DTT. Finally, the samples were heated for 10 min at 95 °C.

### 2.3. Anti-FLAG Immunoprecipitation

At 48 h after transfection with MBP_Flag_TurboID, MBPCit_Flag_TurboID, p21_Flag_TurboID, and Flag_TurboID constructs, transfected HEK293T cells were washed with PBS and lysed in 1 mL TNE buffer (50 mM Tris HCl, pH 8.0, 150 mM sodium chloride, 1% NP40, 1 mM EDTA) supplemented with protease inhibitors (Sigma Aldrich, St. Louis, MO, USA) and 1 mM PMSF for 30 min on ice. After 30 min, cell lysates were sonicated with an ultrasonic homogenizer. To remove insoluble debris, lysates were spun for 20 min at 10,000 g and 4 °C, and the supernatants were passed through the 0, 22-micron syringe filters. The cleared lysates were immediately used for the immunoprecipitation. 1% of the cleared lysates were kept as input controls. The cleared lysate was incubated with 20 μL of anti-FLAG M2 Affinity Gel (Sigma-Aldrich, St. Louis, MO, USA) or Pierce Protein A/G Agarose (Thermo Fisher Scientific, Waltham, MA, USA) slurry at 4 °C for overnight. Following incubation, agarose beads with immunocomplexes were washed with TNE buffer five times, and immunocomplexes were eluted from agarose beads with sample buffer (65.8 mM Tris HCl, pH 6.8, 10% glycerol, 1% SDS, 0.01% bromophenol blue) at 65 °C for 5 min. The supernatants were treated with 5 μL of 2-mercaptoethanol at 95 °C for 5 min. Supernatants containing immunocomplexes were resolved by sodium dodecyl sulfate-polyacrylamide gel electrophoresis (SDS-PAGE), and obtained gels were stained using Coomassie blue.

### 2.4. Immunofluorescence, Image Acquisition, and Analysis in Cell Culture

HEK293T cells were grown on poly-L-lysine–coated coverslips overnight and transfected with expression plasmids for Flag_TurboID, MBP_Flag_TurboID, MBPCit_Flag_TurboID or p21_Flag_TurboID. 4% PFA was used as a fixative solution with a 30 min incubation at room temperature. This was followed by permeabilization with 0.1% Triton-X100 in 1× PBS and blocking with 0.1% Tween-20, 1% BSA and 10% normal goat serum in 1× PBS for 1 h at room temperature. Cells were incubated with primary antibodies anti-FLAG tag (1:1000, F7425, Millipore) overnight at 4 °C. After washing in PBS; the coverslips were incubated with Alexa Fluor 488-conjugated secondary anti-rabbit (1:1000, Invitrogen, A32731) and Streptavidin-SF647 (1:1000, Biotium, 29039) for 1 h at RT. Nuclei were counterstained with Hoechst 33342 (1:5000, Thermo Fisher Scientific, 62249) and the coverslips were mounted on glass slides using ProLong Glass Antifade Mountant (Invitrogen, P36980). For high-resolution imaging, z-series were acquired with an ECLIPSE Ti2 fluorescence microscope (Nikon) equipped with a Spectra III multi-LED light engine (Lumencor), filter cubes for DAPI, FITC, CF647, and ORCA-Fusion BT Digital CMOS camera (Hamamatsu), using NIS Elements software (Nikon). Within each experiment, all groups were imaged with the same acquisition settings. Imaging parameters were set so that the obtained pixel fluorescence intensity was within the dynamic range of the camera to avoid overexposure. Out-of-focus blur was removed from the z-series of fluorescence images via three-dimensional (3D) deconvolution with the NIS-Elements Advanced Research deconvolution package.

### 2.5. Mass Spectrometry Analysis

The strips from the bands stained by Coomassie blue were excised and subjected to a trypsin in-gel digestion procedure. In-gel digestion of protein with trypsin was performed as described previously [40]. After overnight tryptic digestion, the resulting peptides were extracted from the gel blocks. Samples were loaded to in house-made trap column (20 × 0.1 mm), packed with Inertsil ODS3 3 μM sorbent (GL Sciences, Tokyo, Japan), in the mobile loading phase (2% acetonitrile (ACN), 98% H_2_O, 0.1% TFA) at flow rate 10 μL/min and separated in an in house-made [41] fused-silica column (300 × 0.1 mm) packed with Reprosil PUR C18AQ 1.9 (Dr. Maisch, Ammerbuch, Germany) at RT into an emitter made using P2000 Laser Puller (Sutter Instrument, Novato, CA, USA). Reverse-phase chromatography was performed using an Ultimate 3000 Nano LC System (Thermo Fisher Scientific, Waltham, MA, USA), which was connected to the Orbitrap Q Exactive Plus mass spectrometer (Thermo Fisher Scientific, Waltham, MA, USA) via a nanoelectrospray source (Thermo Fisher Scientific, Waltham, MA, USA). As mobile phase A, water containing 0.1% (*v*/*v*) formamide was used, and as mobile phase B, acetonitrile containing 0.1% formamide (*v*/*v*), 20% water (*v*/*v*) was used. Peptides were eluted from the trap column with a linear gradient: 3–6% of B for 3 min; 6–25% of B for 30 min, 25–40% of B for 25 min, 40–60% of B for 4 min, 60% of B for 3 min, 60–99% of B for 0.1 min, 99% B during 10 min, 99–2%B for 0.1 min at 500 nL/min flow rate. After each gradient run, the column was preequilibrated with buffer A for 10 min. MS data were collected in DDA mode. MS1 parameters were the following: resolution—70 K, scan range—350–1500, max injection time—30 s, AGC target—3 × 106. Ions were isolated with a 1.4 *m*/*z* window, preferred peptide match and isotope exclusion. Dynamic exclusion was set to 30 s. MS2 fragmentation was performed in HCD mode at 17.5 K resolution with normalized collision energy (NCE) of 29%, max injection time of 50 s, AGC target—2 × 105, and loop count—10. Other settings were the following: charge exclusion—unassigned, 1, >7 [42,43].

### 2.6. Data Analysis

Raw LC-MS/MS data from the Orbitrap Q Exactive Plus mass spectrometer were converted to mgf peak lists with MSConvert software (ProteoWizard Software Foundation). The following command line parameters were used for this procedure: “--m– --filter peakPicking true [1,2]”. For exhaustive protein identification, obtained peak lists were processed by MASCOT (version 2.5.1, Matrix Science Ltd., London, UK) and X! Tandem (ALANINE, 2017.02.01, The Global Proteome Machine Organization) against the UniProt Knowledgebase (taxon human; downloaded from http://www.uniprot.org accessed on 12 May 2021) with the concatenated reverse decoy database. The precursor and fragment mass tolerance were set at 20 ppm and 50 ppm, respectively, for both search algorithms. The database search parameters were settled as follows: tryptic digestion with one possible missed cleavage, static modification for carbamidometh©(C), and dynamic/flexible modifications for oxidation (M) and biotinylation (K or N-terminal). Selected parameters for X! Tandem allowed for rapid detect protein N-terminal acetylation, peptide N-terminal glutamine ammonia loss or peptide N-terminal glutamic acid water loss. To compare the identification results of MASCOT and X! Tandem and to compile the final list of identified proteins, the resulting files from both search algorithms were subjected to Scaffold 5 (version 5.1.0, Proteome Software Inc, Portland, OR, USA) for validation and further analysis. We used the local false discovery rate scoring algorithm with standard experiment-wide protein grouping. For the evaluation of peptide hits, a false discovery rate (FDR) of less than 1% was selected for peptides and proteins. FDR estimates were based on reverse decoy database analysis.

For further comparative analysis, we used a quantitative assessment based on peptide-to-spectrum matches (PSMs), as we reported previously [26,44,45]. Normalization was carried out on the total number of identified spectra in each sample. No imputation was carried out.

## 3. Results

### 3.1. Study Design

In the current study, we used two isoforms of MBP, the classical 18.5 kDa isoform (P02686) and the variant with reduced basic charge (MBPCit)—analog of C8 isoform [46], detected at a high level, e.g., in the aggressive Marburg variant of MS. It’s recombinant analog contains six arginine amino acid residues of human 18.5-kDa MBP isoform substituted by glutamine residues: Arg 25, 33, 122, 130, and 170 [47]. Additionally, p21 was chosen as a structural control analog of MBP, mimicking it in terms of natively unfolded state, size and basic amino acid clusters. Two different approaches were used to identify the array of proteins interacting with MBP. The first approach involved the enzymatic addition of proximity labels to proteins surrounding the target (TurboID). The second approach represented the classical immunoprecipitation method.

Proximity labeling is carried out using enzymes that catalyze the conversion of an inert low molecular weight substrate into a highly reactive and short-lived diffusible intermediate. This reactive molecule, usually conjugated to an affinity tag, such as biotin, diffuses from the active site of the enzyme and non-specifically covalently labels nearby endogenous biomolecules. For the second method of the protein-protein interaction analysis, the classical immunoprecipitation method was used with a FLAG-tag in a target protein as an affinity label (FLAG-IP).

**Figure 1:**
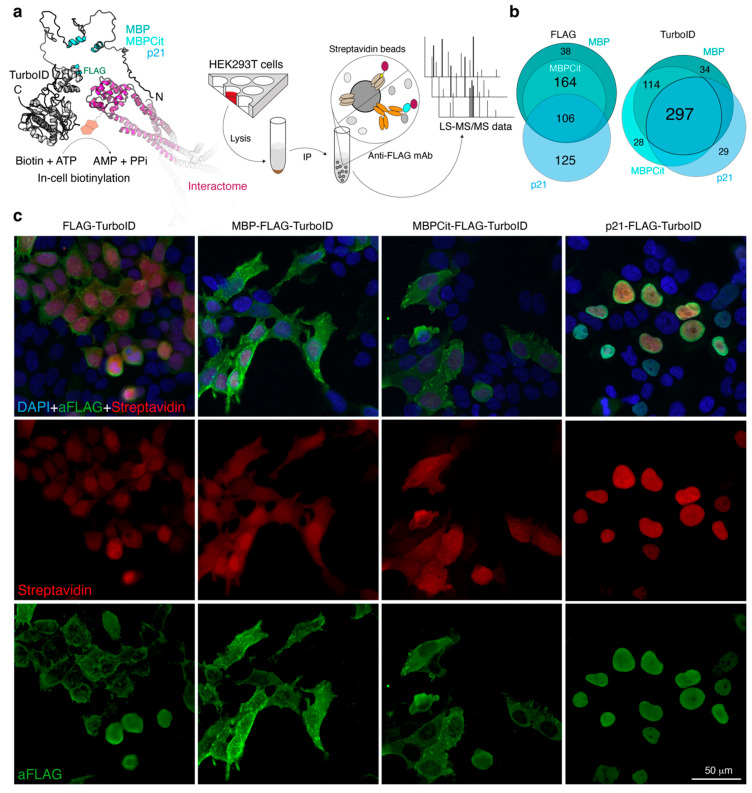
(**a**) General scheme of the experiment. The interactome of MBP, MBPCit and p21 was analyzed by two techniques. The first method included proximity labeling with biotin using the biotin ligase (TurboID) fused with the target proteins. The second method was immunoprecipitation using FLAG-tag and M2-resin. Each method for each protein was carried out in triplicates. The interacting proteins were identified by mass spectrometry-based proteomics with consequent bioinformatic analysis. (**b**) Venn diagram representing proteins identified by TurboID and anti-FLAG-IP methods. (**c**) Localization of FLAG-tagged proteins and biotin in HEK293T cells transfected with Flag-TurboID, MBP-Flag-TurboID, MBPCit-Flag-TurboID and p21-Flag-TurboID expression plasmids. Transfected cells were incubated with anti-FLAG antibodies followed by staining with Alexa Fluor 488-conjugated secondary antibodies against rabbit (green) and streptavidin-SF647 (red). Blue is nuclear staining using Hoechst 33342.

The general scheme of the analysis and the number of identified interactors are shown in Figure 1a,b, respectively. The MBP, MBPCit or p21 were overexpressed in HEK293T cells transfected by constructs, in which the DNA coding for the target protein was followed by FLAG-tag and TurboID biotin ligase sequences. TurboID constructs with the FLAG-tag at the N-terminus were used as control. To initiate biotinylation in the TurboID method, 50 µM biotin was added to the cells for 15 min. Transfected cells were lysed, and protein complexes were precipitated from clarified lysates using either streptavidin beads or an anti-FLAG resin. The resulting protein pools were separated by gradient SDS-PAGE. The gels were stained with Coomassie blue. For mass spectrometry-based proteomics analysis, vertical strips 1 mm wide were cut from the middle of the Coomassie-colored gel bands [26]. The strips were cut into 1 × 1 mm pieces and subjected to the in-gel-trypsinolysis procedure and subsequent MS analysis. The transfection efficiency, localization of target proteins and conjugated biotin in transfected cells were monitored using immunocytochemistry (Figure 1c). As anticipated, TurboID-fused p21 had distinct nuclear localization, whereas MBP fused with TurboID was associated with membrane and cytoplasm.

Bioinformatics analysis of the obtained proteomic data (Supplementary Table S4) is presented in Supplementary Table S1. For each approach (FLAG-IP and TurboID), we identified arrays of proteins, which were differentially increased in the precipitates from MBP-, MBPCit-, or p21-transfected cells compared to the negative control (Flag_TurboID transfection). Proteins were considered differentially increased if they were identified in at least two repeats of the target (non-control) group and their representation exceeded the control by two or more times (Supplementary Table S1, Sheets Biotin and FLAG for TurboID and FLAG-IP methods, respectively).

The overall detected interactomes for each method included about 500 different proteins identified by Mascot and/or X! Tandem with a false discovery rate (FDR) for peptide-spectrum matches less than 0.01 determined by searching a reverse database. Contaminating proteins, such as proteins originating from the skin, serum, cellular response to a viral infection, as well as proteins identified in less than four out of nine samples total for three groups (MBP, MBPCit, and p21), were excluded from the obtained arrays (Sheet Nonspecific of Supplementary Tables S2 and S3 for TurboID and FLAG-IP methods, respectively). At the final stage of the analysis, all selected proteins for each approach were divided into the following groups: common for all three MBP, MBPCit and p21 (Sheets MBP_Cit_p21 in Supplementary Tables S2 and S3), common for MBP and MBPCit (Sheets MBP_Cit in Supplementary Tables S2 and S3), and specific for MBP, MBPCit and p21 alone (Sheets MBP, Cit, and p21 in Supplementary Tables S2 and S3).

Venn diagrams illustrating the distribution of identified proteins in all cohorts were prepared based on data from Supplementary Tables S2 and S3 for TurboID and FLAG-IP methods, respectively (Figure 1b). In the TurboID method, 297 interacting proteins were common to all three target proteins (MBP, MBPCit and p21), 114 proteins were identified as common for MBP and MBPCit, while 34 and 28 proteins were specific to MBP and MBPCit, respectively. In the FLAG-IP method, MBP, MBPCit, and p21 shared 106 proteins, MBP and MBPCit shared 164 proteins, while 38 and zero proteins were MBP- and MBPCit-specific, respectively. Thus, recruiting in the experiment scheme a control protein, p21, resembling MBP in its structural properties, made it possible to exclude from the resulting interactomes 297 and 164 proteins in the case of TurboID and FLAG-IP methods, respectively.

### 3.2. Protein-Protein Interaction Networks Functional Enrichment Analysis of MBP Interactome

**Figure 2:**
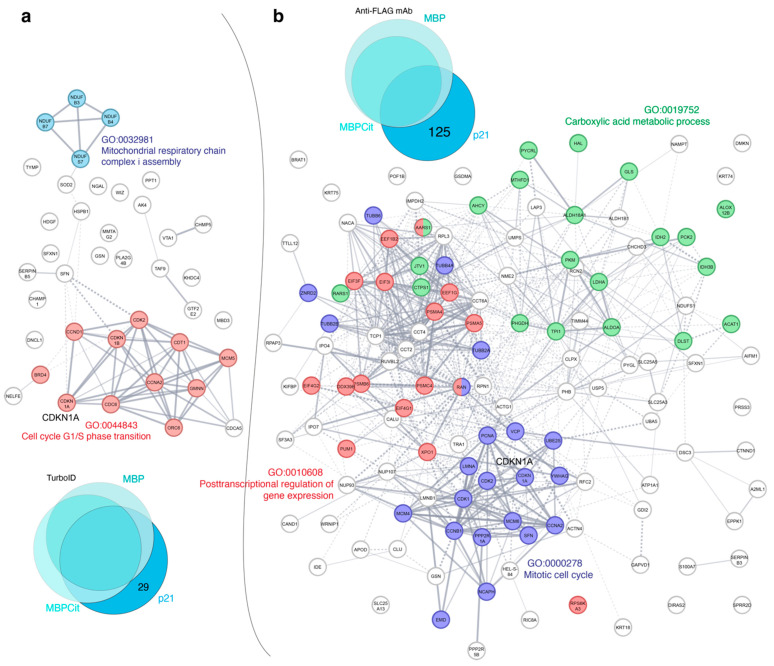
Interaction network between prospective p21 partners identified by TurboID (**a**) and FLAG-IP (**b**) methods. The amount of identified proteins involved in the analysis is shown in the respective Venn diagram. Each color indicates belonging to a specific metabolic pathway of the cell, signed with the same color.

**Figure 3:**
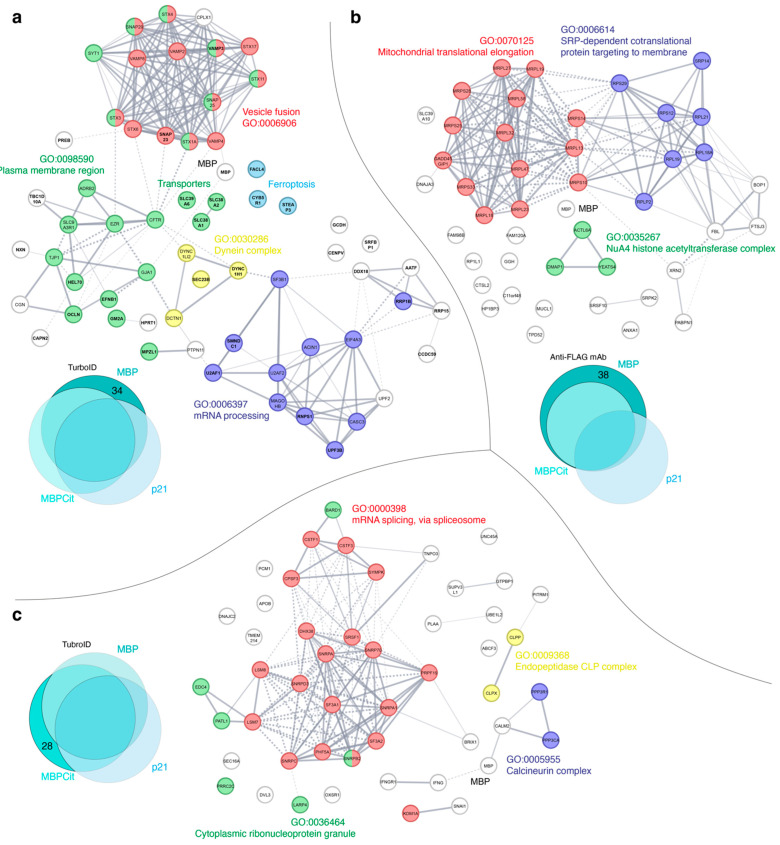
Interaction network between prospective MBP (**a**) and (**b**) and MBPCit (**c**) partners identified by the TurboID (**a**) and (**c**) and FLAG-IP (**b**) methods. The amount of identified proteins involved in the analysis is shown in the respective Venn diagrams. The UniProt identifiers of the interacting proteins on panel (**a**), observed in this work, are shown in bold. Each color indicates belonging to a specific metabolic pathway of the cell, signed with the same color.

Further analysis of the different sets of identified proteins was performed using the STRING database [48,49]. We compared the interaction network reconstructed by the STRING database for proteins identified by two methods (TurboID or FLAG-IP) as specified for each target protein MBP, MBPCit, and p21 (Figure 2 and Figure 3), common for both MBP and MBPCit (Supplementary Figure S1) and common for all three MBP, MBPCit, and p21 (Supplementary Figure S2). Interaction networks specific for individual target proteins and common for MBP and MBPCit clearly distribute to functionally distinct clusters. In contrast, interacting proteins common to all three target proteins, MBP, MBPCit, and p21, did not form distinct functional clusters.

The STRING interaction networks obtained for p21 included 29 and 125 putative interacting proteins identified by the TurboID and FLAG-IP methods, respectively (Figure 2). In accordance with the nuclear localization and the function of p21 as a regulator of cell cycle progression, in both networks, the major functional clusters contained proteins involved either in the cell cycle G1/S phase transition (TurboID method) or in the mitotic cell cycle and posttranscriptional regulation of gene expression (FLAG-IP method). In addition, a small cluster of proteins involved in the assembly of the mitochondrial respiratory chain complex was identified by the TurboID method, and a large set of proteins involved in the metabolism of carboxylic acids was identified by the FLAG-IP method.

The results of similar analysis applied to proteins identified by the two precipitation methods as common partners for both MBP and MBPCit or specific to either MBP or MBPCit are shown in Supplementary Figure S1 and Figure 3, respectively. Interacting proteins identified by classical immunoprecipitation as specific for MBP or common for MBP and its partially discharged counterpart MBPCit constitute three functional clusters according to Gene Ontology classification [50]: mitochondrial translational elongation (GO:0070125), SRP-dependent cotranslational protein targeting to membrane, and RNA processing (GO:0006396). The group of MBP-specific proteins lacks the third cluster; instead, three nuclear proteins from the NuA4 histone acetyltransferase complex (GO:0035267) are present (Figure 3b).

The same Figures show that the arrays of common and specific partners determined by the TurboID method for MBP and MBPCit differ from those identified by the FLAG-IP method. Obviously, the proteins involved in MBP biosynthesis (localized translation) were detected, as well as proteins involved in ribonucleoprotein complexes (GO:1990904), mRNA processing (GO:0006397), cytoplasmic ribonucleoprotein granule (GO:0036464), mRNA splicing via spliceosome (GO:0000398), and in transcription-coupled nucleotide-excision repair (GO:0006283). The proteins from the latter group can apparently be attributed to the case of nonspecific labeling of protein complexes.

In addition to the aforementioned cytoplasmic and nuclear functional clusters, the TurboID method identified a large number of proteins belonging to the plasma membrane region (GO: 0098590), as well as proteins involved in vesicle fusion (GO:0006906), intracellular traffic (transporters), dynein (GO:0030286) and calcineurin (GO:0005955) complexes, cell-cell junction organization (GO:0045216), myelin sheath (GO:0043209), and nervous system development (GO:0007399).

Thus, using classical immunoprecipitation with agarose beads and anti-FLAG antibodies, we mostly observed nuclear and cytoplasmic proteins associated with MBP biosynthesis, which is in accordance with the known phenomenon of localized MBP translation, which will be discussed in detail in the next charter. Neither the membrane nor membrane-associated proteins were observed in the resulting arrays of proteins which were co-precipitated with FLAG-tagged MBP or MBPCit. Comparison of the interaction networks presented in Figure 3 and Supplementary Figure S1 show that in contrast to the FLAG-IP method, the TurboID method results in more heterogeneous arrays, including both membrane-bound proteins and those involved not only in MBP biosynthesis but also in additional cellular processes, which will be discussed in the charters 4.2–4.5. This may be due to intrinsic methodological limitations of the FLAG-IP method, including a mild lysis buffer that does not sufficiently solubilize the membrane components, while the TurboID method does not have this limitation. Opposite to the method of immunoprecipitation, which identifies proteins that directly interact with the target protein, the proximity labeling method identifies proteins positioned in a certain radius from the target protein. This method, while it does not require direct interaction, has the advantage that it eliminates steric problems inherent to the use of antibodies in precipitation and disruption of interactions due to the use of detergent-containing buffers since labeling occurs *in vivo*.

Thus, we may conclude that the TurboID method, although giving a high level of background biotinylation, is a more informative, robust, and reproducible technique compared to the immunoprecipitation, possibly due to higher affinity during the fraction enrichment and easier maintaining of uniform conditions, as well as the manner of interaction being closer to *in vivo*.

## 4. Discussion

MBP, being a major structural protein in myelin, is primarily responsible for the compaction and stabilization of the major dense line. MBP transcription unit of Golli (Gene in the Oligodendrocyte Lineage) gene complex contains seven exons, and due to the alternative splicing of exons 2, 5, and 6 results in five isoforms of myelin basic protein (MBPs) ranging from 14 to 21.5 kDa in size. Expression of splicing isoforms containing the exon-2 sequence (21.5 and 17.2 kDa) has been found to be early-developmental in the initial stages of myelination or remyelination [51,52]. 21.5-kDa isoform is predominantly nuclear-localized, resulting in a promotion of oligodendrocyte proliferation. Expression of exon 2 containing MBP has been considered as a molecular marker of remyelination in a mouse model of allergic encephalomyelitis (EAE) [53], which is consistent with the data that knockout of tyrosine kinase Fyn, which expression is up-regulated during oligodendrocyte progenitors differentiation in primary cultures [54] and declines in active myelinogenesis, drastically decreases 21.5 and 17.2 MBP isoform expression [55]. The 18.5-kDa MBP isoform chosen for this study is predominant in adult myelin and membrane-associated, forming a two-dimensional molecular sieve restricting protein diffusion into compact myelin. Additionally, the high background biotinylation signal of the unfused TurboID (Figure 1c) in the nucleus might significantly increase false positive hits in the case of exon2-containing MBP. Thus, we focused on the 18.5 kDa MBP isoform in the current study.

Indeed, usage of the HEK293T cell line is an evident limitation of our study, which may result in the loss of oligodendrocyte-specific proteins that are absent in HEK293T. Nevertheless, one may formulate several statements reasoning utilization of HEK293T cell line for investigation of MBP interactome: (i) homologs of oligodendrocyte-specific proteins can be identified as MBP interactors in HEK293T cells due to the redundancy of protein expression; (ii) level of transit expression of TurboID-fused proteins in HEK293T cells is almost unreachable in comparison with transduction of primary cell lines; (iii) high level of MBP in cells endogenously overexpressing myelin components may interfere with recombinant TurboID-fused variant, thus, effectively competing for intracellular interactors. The last two points synergistically may result in at least one order of magnitude reduced sensitivity of TurboID proximity labeling proteomics.

All methods of massive detection of the protein environment have their drawbacks. In the case of identifying interactomes, where the target interacting proteins are present in an amount of several orders of magnitude less than the contaminating non-interacting cellular components, this greatly complicates their identification by mass spectrometry. Thus, all approaches to the interpretation of mass spectrometric data are probabilistic, no matter how high the reliability rating is. Although FDR = 1% is a fairly high-reliability rating as of today, there is still a possibility that some identifications were false-positive in reality. To resolve this issue and filter out the proteins assigned as MBP interactors “by mistake”, we specifically introduced in the experiment not only the mock TurboID protein as a negative control to subtract the background of the method but also the p21 protein, which served as a control to subtract proteins randomly identified as interactors during protein translation and processing.

Also, as an additional layer of quality control of identification, we used the classical immunoprecipitation method (IP), which is undoubtedly, a golden standard for validating data obtained from proteomic studies. However, it should be noted that TurboID is much more sensitive in comparison with ordinary IP. Moreover, TurboID proximity labeling allows detecting cryptic interaction, e.g., membrane-associated interactome, which is especially important in the case of unstructured and membrane-trapped MBP molecules. In frames of the current study, we used IP together with TurboID proximity labeling (Figure 3a,b), and a significant number of the hits were different. Additionally, we previously reported results of the cross-linked MBP IP [26]. Indeed, many MBP partners involved in membrane adhesion and cytoskeleton organization identified by both methods are the same. However, these cohorts do not have to be completely overlayed.

### 4.1. MBP Interactors Involved in the mRNA Processing and Maintenance

Localized MBP translation is common for oligodendrocytes MBP is transported in oligodendrocytes in the form of mRNA rather than as a protein [56]. In accordance with this theory, we have discovered a large pool of proteins that regulate the processes of protein biosynthesis, starting with RNA processing (Figure 3). Heterogeneous nuclear ribonucleoproteins (hnRNPs) are a complex of RNA and protein present in the cell nucleus during gene transcription and subsequent post-transcriptional modification of newly synthesized RNA (pre-mRNA). The presence of proteins associated with the pre-mRNA molecule serves as a signal that the pre-mRNA is not yet fully processed and, therefore, is not ready for export to the cytoplasm [57].

The hnRNPs are also an integral part of the 40S subunit of the ribosome and hence are important for mRNA translation in the cytoplasm. However, hnRNPs are found primarily in the nucleus as they have their own nuclear localization sequences (NLS), and their main role is to bind to newly transcribed RNAs. hnRNPs are also responsible for the enhancement and inhibition of splicing sites, making such sites more or less accessible to the spliceosome [58]. Cooperative interactions between attached hnRNPs can stimulate certain splicing combinations and inhibit others [59]. MBP has been found to interact with hnRNP A3 and hnRNP Q. Very little is known about the function of hnRNP A3, but it is unusual that in addition to being involved in protein biosynthesis, hnRNP A3 has been shown to promote aberrant nuclear localization of EGFR [60]. HnRNP Q has been found to be involved in many mRNA maturation steps [61]. Interestingly, hnRNP Q promotes the development of dendrites and the formation of focal adhesion in neurons [62]. Knockdown of hnRNP Q in mouse cortical neurons shows an increase in the length of axons and neurites [63].

Other nuclear proteins found among MBP interactors, DEAD box containing putative RNA helicases, are characterized by the conserved motif Asp-Glu-Ala-Asp (DEAD) named after the amino acids of motif II or Walker B (Mg2+-binding aspartic acid). They are implicated in several cellular processes involving alteration of RNA secondary structure, such as translation initiation, nuclear and mitochondrial splicing, and ribosome and spliceosome assembly. In addition, DEAD-box proteins have important roles in RNA metabolism, from RNA transcription to degradation, such as RNA transport, ribosome biogenesis, translation and RNA decay [64,65]. These enzymes unwind double-stranded RNA molecules in an energy-dependent fashion through the hydrolysis of NTP. DEAD-box RNA helicases belong to superfamily 2 (SF2) of helicases. Like other SF1 and SF2 members, they contain seven conserved motifs, which are characteristic of these two superfamilies [66]. The RNA helicase DeaD is ATP-dependent [67] and is induced by low temperatures [68]. In addition to its role in unwinding double-stranded RNA, it is involved in ribosomal subunit biogenesis [69]. A number of DEAD boxes containing RNA helicases were found among proteins interacting with MBP, in particular, DDX1, DDX3X, DDX17, and DDX21. Previously, DEAD box containing RNA helicases was shown to be actively involved in the regulation of myelin biosynthesis. Ddx20 dead box protein suppresses the transcriptional activity of Egr2, which is a master regulator of myelination [70]. Another DEAD box-containing RNA helicase, DDX54, was shown to localize with MBP in oligodendrocyte lineage cells and to be crucial for the myelinization process in CNS [71,72]. As well as DDX5 is involved in the post-transcriptional regulation of MBP protein synthesis, with implications for oligodendroglial development [73].

### 4.2. MBP Interactors—Members of Protein Synthesis Machinery

Another two large groups of proteins associated with MBP (or MBPCit) biosynthesis are proteins involved in the elongation of translation, including mitochondrial translation, and in SRP-dependent cotranslational protein targeting to the membrane (Figure 3). We have previously shown the association of MBP with proteins from the translational machinery [26], and now these data are additionally confirmed. The importance of the association of MBP with mitochondria has been shown previously [74,75,76]. SRP-dependent cotranslational protein targeting to the membrane occurs during translation and is dependent upon two key components, the signal-recognition particle (SRP) and the SRP receptor. SRP is a cytosolic particle that transiently binds to the endoplasmic reticulum (ER) signal sequence in a nascent protein and to the SRP receptor in the ER membrane [77]. Detection of SRP-associated proteins in the MBP environment may be due to the fact of sharing the interaction with calmodulin, functioning as a chaperon for the ER secretory pathway [78]. In this regard, we must note that among the proteins interacting with MBPCit, two proteins involved in Ca^2+^ signaling were found, namely calmodulin 2 (*CALM2*) and protein phosphatase 3 catalytic subunit alpha (*PPP3CA*). CALM2 is a member of the calmodulin family [79]. It is a calcium-binding protein that plays a role in signaling pathways, cell cycle progression and proliferation [80]. PPP3CA is a catalytic subunit of calcineurin (CN) [81] and highly conserved Ca^2+^/calmodulin-activated Ser/Thr phosphatase. PPP3CA is ubiquitously expressed and is particularly abundant in the brain. By dephosphorylating a variety of protein substrates in response to Ca^2+^ signals, CN regulates development, learning and memory, cardiac function, and the immune response [81]. One of the best-studied activities of CN is its dephosphorylation of the nuclear factor of the activated T cell family of transcription factors (NFATc1-c4), which allows NFAT to translocate to the nucleus where it induces the expression of genes required for T cell activation [82]. Nfat/calcineurin signaling has previously been shown to promote oligodendrocyte differentiation and myelination [83].

### 4.3. MBP Interactors Associated with Cellular Adhesion and Transmembrane Traffic

As we mentioned here previously, the employment of the proximity labeling method (TurboID) allowed us to significantly expand the MBP interactome by discovering in the MBP environment proteins involved not only in the MBP biogenesis but in the additional cellular processes, such as adhesion. We have identified moesin (*MSN*), which is involved in the formation of bonds between the membrane and the cytoskeleton [84]. The moesin paralog, ezrin, is specifically expressed in Schwann cells, where it maintains the integrity of the myelin sheath [85,86]. In particular, we found occludin, an integral membrane protein with four transmembrane domains that is exclusively localized at tight junctions [87,88], and Ephrin-B1, which belongs to the subfamily Ephrins-Bs (Ephrin-B1 to B6). Ephrins-Bs are type I membrane proteins and ligands of Eph-related receptor tyrosine kinases [89,90], which are crucial for migration, repulsion and adhesion during neuronal, vascular and epithelial development. The Eph-ephrin receptor system is an important mediator of bidirectional signaling between axons and oligodendrocytes. In the process of selecting axons for myelination, the combination of Eph-ephrin forward and backward signaling is important. In particular, the ephrin-B reverse signaling induced by EphA4 or EphB1 enhances the formation of myelin sheets [91]. We also found Myelin protein zero-like protein 1 (*MPZL1/PZR*). MPZL1 is a single transmembrane glycoprotein that is involved in extracellular matrix-induced signal transduction [92,93]. MPZL1 has an important role in cell signaling via c-Src as a major receptor of concanavalin A [94].

Among the membrane-associated proteins specific to MBP, a neuronally-expressed solute carrier (SLC) for glutamine, Sodium-coupled neutral amino acid transporter 1 (SNAT1), which belongs to a subfamily of proteins that show structural characteristics of zinc transporters [95], was also found. The SLC group of membrane transport proteins includes over 400 members organized into 66 families [96,97]. SNAT1 is an important transporter of glutamine, which serves as a precursor for the synaptic transmitter glutamate [98].

### 4.4. MBP Interactors Associated with Lipid Metabolism and Ferroptosis

Interestingly, among the proteins interacting with MBP, we identified proteins involved in ferroptosis. Namely, they are long-chain-fatty-acid--CoA ligase 4 (ACSL4, *FACL4*), NADH-cytochrome b5 reductase 1 (*CYB5R1*) and metalloreductase STEAP3 (*STEAP3*). ACSL4 catalyzes the conversion of long-chain fatty acids to their active form, acyl-CoA, for both synthesis of cellular lipids and degradation via beta-oxidation [99]. *FACL4* gene is highly expressed in the brain and has been shown to be involved in nonspecific intellectual disability and fatty-acid metabolism [100]. STEAP3 is an endosomal ferrireductase capable of converting iron from an insoluble ferric (Fe^3+^) to a soluble ferrous (Fe^2+^) form [101]. Ferroptosis, distinct from necrosis, autophagy and apoptosis, is a unique form of regulated cell death and is a potential pathogenic mechanism of neuronal loss and dysfunction in many neurodegenerative disorders. Recent studies indicate that oligodendrocytes are especially sensitive to lipid peroxidation, among which independent lipid peroxidation is an essential feature of ferroptosis [102]. Recent studies have shown the presence of iron deposition in the central nervous system (CNS) of patients with multiple sclerosis (MS) [103]. In addition, we found a Ganglioside GM2 activator (GM2A), which binds gangliosides and stimulates ganglioside GM2 and glycolipid GA2 degradation by beta-hexosaminidase A [104].

### 4.5. MBP Interactors Involved in Vesicular Fusion and Trafficking to Plasma Membrane Region

Another important group of MBP-associated proteins found in this study includes molecules involved in vesicular fusion and trafficking to the plasma membrane region. SEC23, the core component of the coat protein complex II (COPII) vesicles, transports newly synthesized proteins and lipids from ER to the Golgi apparatus for secretion [105,106]. Rab2 belongs to the Rab family of small guanosine triphosphatases (GTPases) that contain highly conserved domains involved in GTP binding and hydrolysis. The Rabs are residents of pre-Golgi intermediates and are required for protein transport from ER to the Golgi complex [107,108]. Rab2 was shown to increase neuronal adhesion and neurite growth in vitro [109]. TBC1 (Tre2/Bub2/Cdc16) domain family member 10A (TBC1D10A), which was also found to be specific for MBP, acts as a GTPase-activating protein for RAB27A [110]. In the MBP interacting group, we found cytoplasmic dynein 1 heavy chain 1 protein from the dynein complex, which has been identified in our previous study [26].

Probably the most important group of identified MBP-associated proteins belongs to the vesicle fusion group (Figure 3). We found the association of MBP with Vesicle-associated membrane protein 3 (*VAMP3*), Syntaxin-binding protein 3 (*STXBP3*) and Synaptosomal-associated protein (*SNAP23*). Vesicular traffic is essential for cellular homeostasis. In general, intracellular protein transport involves the liberation of cargo-containing transport vesicles from “donor” membranes and the subsequent docking and fusion of these vesicles with the target or “acceptor” membranes [111]. It is evident that vesicle docking and vesicle fusion are distinct processes mediated by distinct proteins (reviewed in Refs. [112,113]). Since the general membrane fusion machinery (consisting of N-ethylmaleimide Sensitive Factor (NSF) and SNAPs) nonspecifically catalyzes membrane fusion, the regulation of fusion between transport vesicles and specific acceptor membranes is thought to lie in the vesicle docking process. In the brain, synaptic vesicle docking is regulated in part by specific interactions of the synaptic vesicle protein synaptobrevin (also known as vesicle-associated membrane protein or VAMP) with the presynaptic plasma membrane-associated proteins syntaxin and SNAP-25 (synaptosome-associated protein of 25 kDa; not related to the SNAPs for NSF). Together these molecules form a stable complex that also functions as a SNAP receptor (“SNARE”). It is believed SNAPs and NSF bind to the SNARE complex at the transport vesicle/target membrane interface so that following vesicle docking membrane fusion can occur.

A general model of protein transport in all cells, the SNARE hypothesis, proposes that the specificity of a particular transport step is regulated by the specific interaction of distinct VAMPs and syntaxins on transport vesicles and target (acceptor) membranes, respectively [114]. There is considerable experimental evidence to support the SNARE hypothesis, including the demonstration that (a) different isoforms of syntaxin and VAMP exist, some of which can be localized to unique intracellular compartments [114,115,116]; (b) that these proteins are often present in multiple tissues in the same organism [115,116,117]; and (c) that a given VAMP isoform is capable of interacting with some, but not all, syntaxins [118]. In addition, homologs of these molecules have been found in yeast, and deletion of the yeast VAMP, syntaxin, or SNAP-25 homologs leads to severe defects in protein secretion (reviewed in Ref. [119]).

Despite the overwhelming evidence supporting the SNARE hypothesis, it has been surprising that SNAP-25 has not been detected in most non-neuronal mammalian tissues [120,121,122]. This is especially true given recent data demonstrating that SNAP-25 is an essential component of the high-affinity general fusion machinery binding site [123]. It has also been shown recently that SNAP-25 increases the affinity of some VAMP-syntaxin interactions but not others [124,125], suggesting that SNAP-25 itself helps in regulating the specificity of transport vesicle docking. SNAP-23, a ubiquitously expressed homolog of SNAP-25, can bind with high affinity to both VAMPs and syntaxins and appears to fulfill the role of SNAP-25 in regulating transport vesicle docking and fusion in all mammalian cells [126].

VAMP3 (known as cellubrevin) is ubiquitously expressed and participates in regulated and constitutive exocytosis as a constituent of secretory granules and secretory vesicles [127]. Cellubrevin is a member of the synaptobrevin/VAMP family of SNAREs, which has a broad tissue distribution. The expression of MBP has been revealed to depend on the correct functioning of the SNARE machinery, which is not required for mRNA granule assembly and transport per se [128]. In a recently published paper by Lam et al., VAMP2/3-mediated membrane expansion in oligodendrocytes is indispensable for myelin formation due to the incorporation of axon-myelin adhesion proteins that are collectively required to form nodes of Ranvier [129].

## 5. Conclusions

In the current study, we exhaustively probed the protein environment of MBP and its uncharged form MBPCit using two fundamentally different methods, as well as using a control protein for additional verification of the specificity of the identified protein partners. We have shown that in the case of low molecular weight unstructured proteins, such as MBP, in vitro methods, which use antibodies that may partially overlap a part of the protein and change its conformation, as well as buffers that change the original cellular environment, may be less informative compared to the methods of identifying the protein environment *in vivo*, such as proximity labeling methods, for example, TurboID. It has also been shown that recruiting a protein that is similar in structural characteristics but differs in function can allow for the exclusion of a pool of proteins identified due to non-specific interactions.

**Figure 4:**
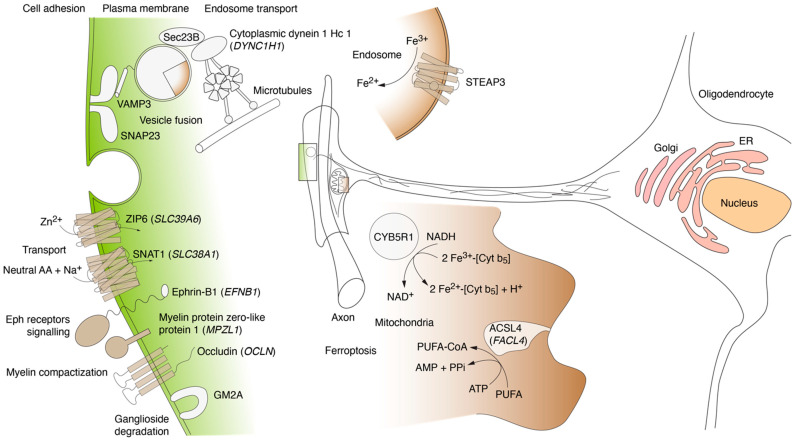
Schematic map illustrating the cellular localization of proteins found by the TurboID method in the immediate environment of MBP, excluding those involved in localized translation.

Figure 4 summarizes the cellular localization of proteins found by the TurboID method in the immediate environment of MBP. The map does not include proteins identified due to their involvement in MBP biosynthesis (localized translation) since this process has already been described in detail and mapped in [56]. The MBP environment includes adhesion proteins occludin and myelin protein zero-like protein 1, solute carrier family transporters ZIP6 and SNAT1, Eph receptors ligand Ephrin-B1, as well as structural components of the vesicle transport machinery synaptosomal-associated protein 23 (SNAP23), vesicle-associated membrane protein 3 (VAMP3), protein transport protein hSec23B and cytoplasmic dynein 1 heavy chain 1. In addition, MBP colocalizes with proteins involved in Fe^2+^ and lipid metabolism, namely, ganglioside GM2 activator protein, long-chain-fatty-acid-CoA ligase 4 (ACSL4), NADH-cytochrome b5 reductase 1 (CYB5R1) and metalloreductase STEAP3. The latter finding suggested that MBP can recruit and regulate the activity of these factors, which is consistent with both the inclusive role of MBP in the integrity of the myelin sheath and the emerging role of ferroptosis in the development of autoimmune neurodegenerations associated with impaired myelination.

## Data Availability

The mass spectrometry proteomics data have been deposited to the ProteomeXchange Consortium via the PRIDE partner repository with the dataset identifier PXD039467 and 10.6019/PXD039467.

## Supplementary Materials

The following are available online at https://www.mdpi.com/article/10.3390/cells12060944/s1, Table S1. Summary table of identified proteins. Table S2. Summary table of proteins identified by TurboID method. Table S3. Summary table of proteins identified by FlagIP method. Table S4. Summary of the identification of proteins and peptides. Figure S1. String interaction network for interactors common for MBP and MBPCit for TurboID and FlagIP methods. Figure S2. String interaction network for interactors common for MBP/MBPCit/p21 for TurboID and FlagIP methods.
