# Supplementary material for: Identification of Myelin Basic Protein Proximity Interactome Using TurboID Labeling Proteomics
Source: Cells. 2023 Mar 20;12(6):944. doi: 10.3390/cells12060944 (PMC10047773; doi:10.3390/cells12060944)
Supplement: Supplementary file 1 [file cells-12-00944-s001.zip › Figure_S2.pdf]

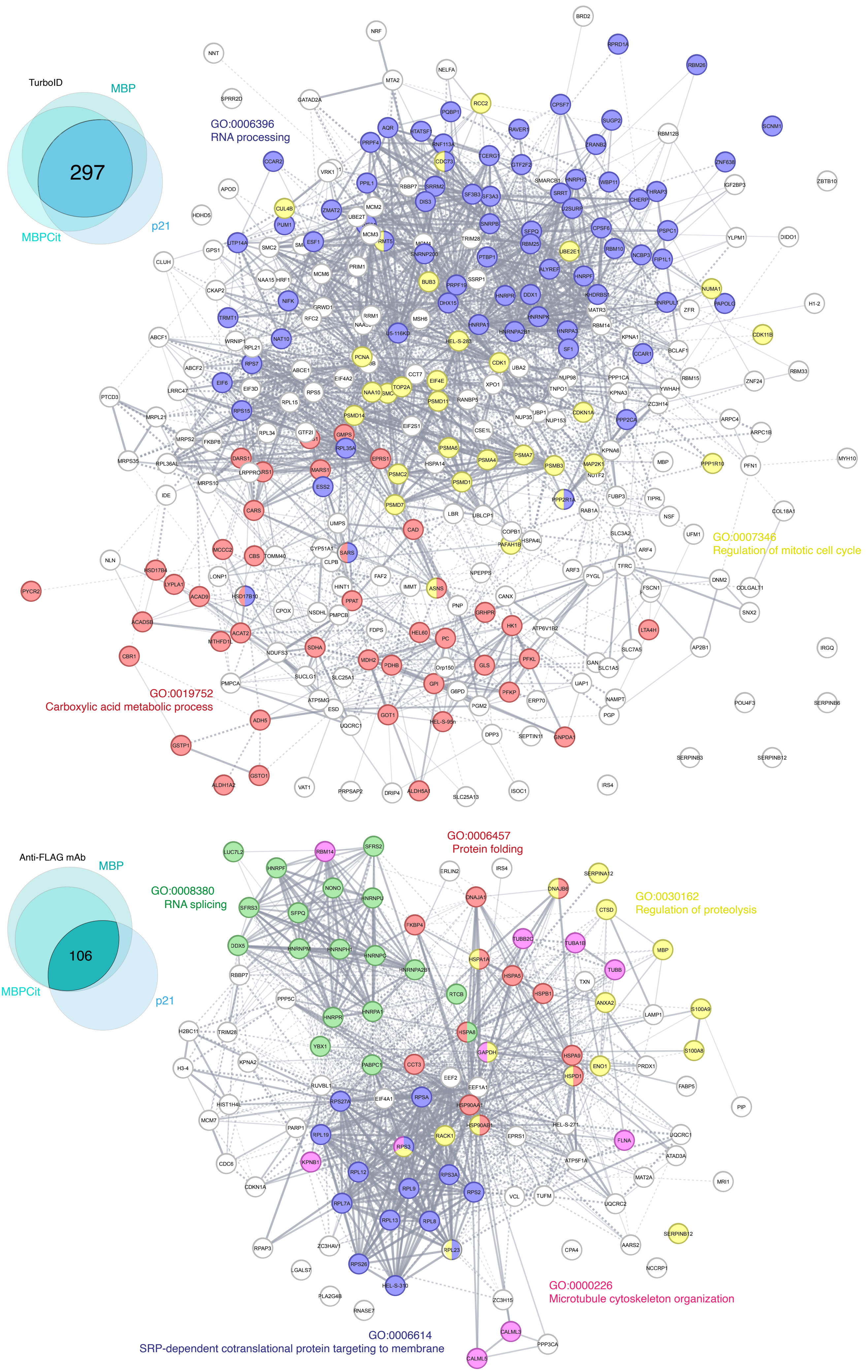

Figure S2. Interaction network between common interactors for MBP, MBPCit, and p21 identified by TurboID (top) and FLAG-IP (bottom) methods. The amount of identified proteins involved in the analysis is shown in the respective Venn diagram. Each color indicates belonging to a specific metabolic pathway of the cell, signed with the same color.
